# Transient dynamics and rhythm coordination of inferior olive spatio-temporal patterns

**DOI:** 10.3389/fncir.2013.00138

**Published:** 2013-09-05

**Authors:** Roberto Latorre, Carlos Aguirre, Mikhail I. Rabinovich, Pablo Varona

**Affiliations:** ^1^Grupo de Neurocomputación Biológica, Dpto. de Ingeniería Informática, Escuela Politécnica Superior, Universidad Autónoma de MadridMadrid, Spain; ^2^BioCircuits Institute, University of California San DiegoLa Jolla, CA, USA

**Keywords:** spike wave fronts, subthreshold oscillations, electrical coupling, multifunctional neural networks, cerebellar circuits, source-sink phenomena, rhythm coordination and encoding, activity reverberation

## Abstract

The inferior olive (IO) is a neural network belonging to the olivo-cerebellar system whose neurons are coupled with electrical synapses and display subthreshold oscillations and spiking activity. The IO is frequently proposed as the generator of timing signals to the cerebellum. Electrophysiological and imaging recordings show that the IO network generates complex spatio-temporal patterns. The generation and modulation of coherent spiking activity in the IO is one key issue in cerebellar research. In this work, we build a large scale IO network model of electrically coupled conductance-based neurons to study the emerging spatio-temporal patterns of its transient neuronal activity. Our modeling reproduces and helps to understand important phenomena observed in IO *in vitro* and *in vivo* experiments, and draws new predictions regarding the computational properties of this network and the associated cerebellar circuits. The main factors studied governing the collective dynamics of the IO network were: the degree of electrical coupling, the extent of the electrotonic connections, the presence of stimuli or regions with different excitability levels and the modulatory effect of an inhibitory loop (IL). The spatio-temporal patterns were analyzed using a discrete wavelet transform to provide a quantitative characterization. Our results show that the electrotonic coupling produces quasi-synchronized subthreshold oscillations over a wide dynamical range. The synchronized oscillatory activity plays the role of a timer for a coordinated representation of spiking rhythms with different frequencies. The encoding and coexistence of several coordinated rhythms is related to the different clusterization and coherence of transient spatio-temporal patterns in the network, where the spiking activity is commensurate with the quasi-synchronized subthreshold oscillations. In the presence of stimuli, different rhythms are encoded in the spiking activity of the IO neurons that nevertheless remains constrained to a commensurate value of the subthreshold frequency. The stimuli induced spatio-temporal patterns can reverberate for long periods, which contributes to the computational properties of the IO. We also show that the presence of regions with different excitability levels creates sinks and sources of coordinated activity which shape the propagation of spike wave fronts. These results can be generalized beyond IO studies, as the control of wave pattern propagation is a highly relevant problem in the context of normal and pathological states in neural systems (e.g., related to tremor, migraine, epilepsy) where the study of the modulation of activity sinks and sources can have a potential large impact.

## 1. Introduction

**Figure 1:**
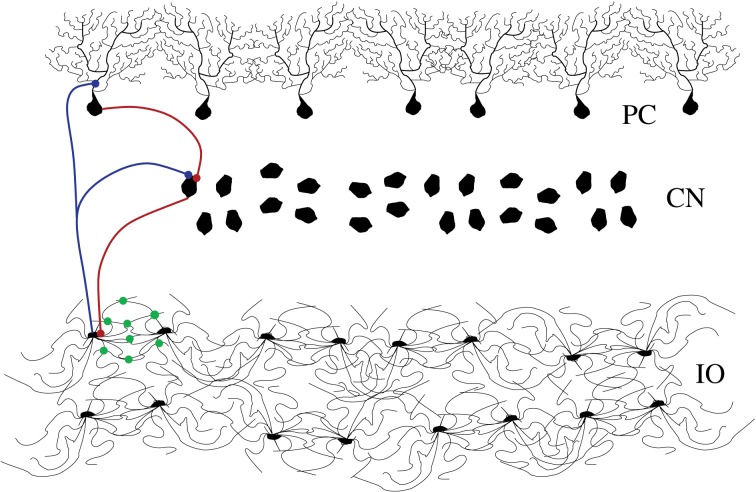
**Schematic representation of the cerebellar inhibitory loop**. The figure shows the inferior olive neurons (IO layer), cerebellar nuclei (CN layer) and *Purkinje* cells (PC layer). Blue connections are excitatory and red ones are inhibitory. The IO neurons are highly coupled with electrotonic synapses (green dots in the figure).

The architecture of the inferior olive (IO) network and the associated circuits of the cerebellar cortex of mammals have been investigated anatomically and physiologically in great detail (De Zeeuw et al., 1998; D'Angelo et al., 2011; De Zeeuw et al., 2011). Experimental recordings, both *in vitro* and *in vivo*, show that IO cells are electrically coupled and display a characteristic behavior with subthreshold oscillations (Llinás and Yarom, 1981; Benardo and Foster, 1986; Lampl and Yarom, 1993; Bal and McCormick, 1997; Long et al., 2002; Chorev et al., 2007; Choi et al., 2010) and spiking activity (Llinás et al., 1974; Sotelo et al., 1974). Their axons transmit synchronous and rhythmic excitatory synaptic input to both the deep cerebellar nuclear cells (CNs) and to the *Purkinje* cells (PCs) of the cerebellar cortex (Uusisaari and De Schutter, 2011). The phasic response of the PCs is transmitted as inhibitory inputs to the CNs. Thus, the nuclear cells are excited by the IO neurons and later inhibited by the PCs. This inhibition leads to rebound excitation. The nuclear cells also send an inhibitory feedback to the IO closing this inhibitory loop (IL) (De Zeeuw et al., 1997; Uusisaari and De Schutter, 2011) (see Figure 1).

While much is now known from the anatomical and physiological perspective, the functional role of the IO is still under discussion (Welsh et al., 1995; Llinás et al., 1997; De Zeeuw et al., 1998; Kobayashi et al., 1998; Kistler and De Zeeuw, 2002; Long et al., 2002; Schweighofer et al., 2004; Dean et al., 2010; Bazzigaluppi et al., 2012). It has been proposed as a system that controls and coordinates different rhythms through the intrinsic oscillatory properties of the individual IO neurons and their electrical interconnections (Llinás and Yarom, 1986; Llinás and Welsh, 1993; Manor et al., 1997; De Zeeuw et al., 1998; Hutcheon and Yarom, 2000; Leznik and Llinás, 2005) by means of clusters of functionally interconnected cells. The IO has also been suggested to be implicated in learning (Ito, 1982; Raymond et al., 1996; Ito, 2005; Van Der Giessen et al., 2008; Schweighofer et al., 2013), in comparing tasks of intended and achieved movements as a generator of error signals (Oscarsson, 1980; Llinás, 2009; Schlerf et al., 2012; Ito, 2013), and as a dynamical working memory in the context of the olivo-cerebellar closed-loop (Kistler and De Zeeuw, 2002).

Electrical gap junctions allow the synchronization of the subthreshold oscillations among groups of neurons (Bennett and Zukin, 2004; Connors and Long, 2004), and through these, the synchronization of the spiking activity. Subthreshold oscillations must clearly have a relevant role for information processing in the context of a system with extensive electrical coupling. In such systems, not only the spiking activity can be propagated through the network, but also small voltage differences of hyperpolarized membrane potentials among neighbor cells. Subthreshold oscillations are present in many other neural systems (Leung and Yim, 1991; Gutfreund et al., 1995; Pape et al., 1998; Amir et al., 2002; Reboreda et al., 2003; D'Angelo et al., 2009), as well as gap-junctions (Bennett and Zukin, 2004; Long et al., 2004). However, the IO seems to be a system where the joint presence of these two features can have a special significance for its function. Several theoretical models of the IO network have been proposed to study its properties and behavior (e.g., see Manor et al., 1997; Varona et al., 2002; Velarde et al., 2004; Jacobson et al., 2008; Katori et al., 2010; De Gruijl et al., 2012; Torben-Nielsen et al., 2012). These studies largely contribute to the understanding of the IO function from a network dynamics perspective.

In this paper we show that large scale networks of electrically coupled IO neurons generate localized spatio-temporal patterns which can easily encode several coexisting rhythms. In our simulations, we have used conductance-based neurons to generate subthreshold oscillations as well as spiking activity in the amplitude and frequency ranges reported for these neurons. We argue that neither the knowledge of the anatomic organization of these neural circuits, nor the study of the individual activity of the cells alone is enough for the identification of their function. However, understanding the collective dynamics gives us important clues about the underlying computational properties and the possible multifunctional nature of the IO. We want to emphasize here the combination of the specific organization of the connections and of the specific dynamics of the neurons as an essential step in understanding the role of the olivo-cerebellar circuits. The use of large population networks of realistic neurons is required for the study of the IO network dynamics and, in particular, the encoding and control of rhythms in the IO transient spatio-temporal activity.

Our results show that the two principal characteristics of the IO, i.e., the subthreshold oscillations of the individual neurons and the electrical gap junctions, make this system a powerful encoder and generator of spatio-temporal patterns with different but coordinated oscillatory rhythms. In our study, we first analyze the factors that shape the patterns in autonomous networks. We show that networks of spiking neurons that do not generate subthreshold oscillations have a more restricted ability to develop dynamical patterns. Then, we study the ability of IO networks to generate and encode reverberating rhythms depending on external stimuli. We also show that the presence of low and high excitability regions in this system creates activity sinks and sources that shape the propagation of coordinated spike wave fronts. Finally, we discuss the modulatory effect that the IL can have on the IO spatio-temporal activity.

## 2. Models and methods

### 2.1. Neuron model

**Figure 2:**
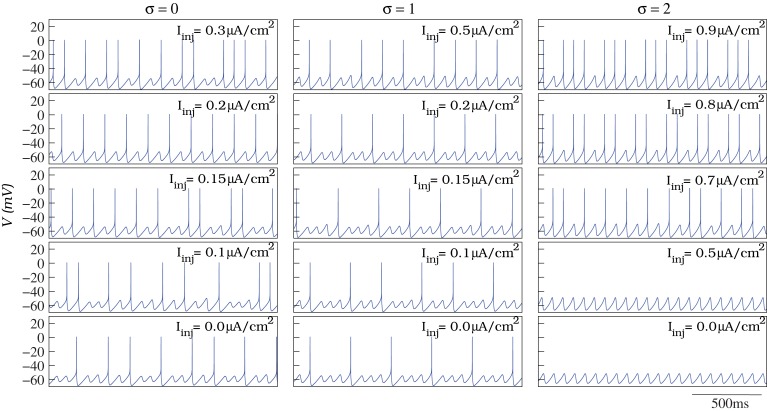
**In our simulations, we model the individual behavior of the single IO cells with a Hodgkin–Huxley type model**. The model is able to generate subthreshold oscillations and spiking activity at different frequencies as a function of the parameters (see the text for details). The figure illustrates different examples of spiking frequencies over the subthreshold oscillations depending on the values of σ (defining the action potential threshold of the model) and *I*_inj_ (constant depolarizing current in Equation 1).

To model the individual behavior of each IO cell, we have built a conductance-based neuron using a Hodgkin–Huxley type formalism (Hodgkin and Huxley, 1952) that generates the characteristic subthreshold oscillations and spiking activity in the amplitude and frequency ranges observed in the living IO cells. Figure 2 shows some examples of the different behaviors displayed by the neuron model as a function of its parameters: from subthreshold oscillations to tonic spiking. The results discussed in this paper do not depend on whether individual neurons are intrinsic or network oscillators (Manor et al., 1997; Schweighofer et al., 1999; Manor et al., 2000; Kistler and De Zeeuw, 2002).

Our model is based on Wang's work on subthreshold membrane potential oscillations in cortical pyramidal cells (Wang, 1993). The model uses a single compartment to describe the neuron dynamics. Since we want to build large scale networks of thousands of units, the computational cost is an important issue. Five voltage-dependent ionic currents (*I*_Na_, *I*_Nap_, *I*_Kd_, *I*_Ks_ and *I*_*h*_), a leakage current (*I*_*l*_) and a stimulus injected current (*I*_inj_) define the specific behavior of the neuron (see below). Formally, the membrane voltage is described by the following equation:

(1) $$C_{m}\frac{dV}{dt}=-(I_{\text{Na}}+I_{\text{Nap}}+I_{\text{Kd}}+I_{\text{Ks}}+I_{h}+I_{l}+I_{\text{inj}}+I_{\text{elec}}+I_{\text{syn}})$$

where *C*_*m*_ = 1 μF/cm^2^; *I*_*l*_ = *g*_*l*_(*V* − *V*_*l*_) with *g*_*l*_ = 0.1 ms/cm^2^ and *V*_*l*_ = −60 mV; and *I*_inj_ is a constant depolarizing current. Currents *I*_elec_ and *I*_syn_ are, respectively, the total current from the electrical gap junctions connecting the neurons to build the IO network and the total synaptic current from the IL (see section 2.2 for a detailed description of these two currents).

The general description of the five active ionic currents considered in the model follows the Hodgkin–Huxley formalism:

(2) $$I_{i}={\bar{g}}_{i}\cdot x^{p}\cdot y^{q}\cdot (V-V_{i})$$

where ḡ_*i*_ is the maximal conductance of the current, *V* is the membrane potential, *V*_*i*_ is the reversal potential of the current and *x* and *y* are the activation and inactivation variables. Table 1 provides the specific values of these parameters for each particular current. The activation and inactivation variables, when exist, satisfy the following equations:

(3) $$\frac{dx}{dt}=\frac{x_{\infty }-x}{\tau_{x}},\;\frac{dy}{dt}=\frac{y_{\infty }-y}{\tau_{y}}$$

**Table 1:** **Conductance description, maximal conductances and reverse potential (Equation 2) for the ionic currents of the single neuron model**.

**Current (μA/**cm**^**2**^)**	**Conductance**	**ḡ_*i*_ (mS/cm^2^)**	***V*_*i*_ (mV)**
*I*_Na_	ḡ_Na_*m*^3^_∞_*h*	ḡ_Na_ = 52	*V*_Na_ = 55
*I*_Nap_	ḡ_Nap_*n*_∞_	ḡ_Nap_ = 0.1	*V*_Na_ = 55
*I*_Kd_	ḡ_Kd_*c*^4^	ḡ_Kd_ = 20	*V*_*K*_ = −90
*I*_Ks_	ḡ_Ks_*d*(ρ*e* + (1 − ρ)*f*)	ḡ_Ks_ = 14	*V*_*K*_ = −90
*I*_*h*_	ḡ_*h*_*t*	ḡ_*h*_ = 0.1	*V*_*h*_ = −43

The steady state and time constants of these variables for each current are:

• ***I***_**Na**_

$$\begin{array}{l} m_{\infty }=\frac{\alpha_{m}}{\alpha_{m}+\beta_{m}};\;\tau_{m}=\frac{1}{\alpha_{m}+\beta_{m}}\\ \alpha_{m}=0.1(V+30-\sigma )\text{​}/\text{​}(1-\exp(-0.1(V+30-\sigma )));\\ \beta_{m}=4\exp((-V-55+\sigma )\text{​}/\text{​}18);\\ h_{\infty }=\frac{\alpha_{h}}{\alpha_{h}+\beta_{h}};\;\tau_{h}=\frac{1}{\alpha_{h}+\beta_{h}}\\ \alpha_{h}=1.99\exp((-V-44+\sigma )\text{​}/\text{​}20);\\ \beta_{h}=\frac{28.57}{1+\exp(-0.1(V+14-\sigma )))}; \end{array}$$

• ***I***_**Nap**_

$$n_{\infty }=\Gamma (V,51,5);$$

• ***I***_**Kd**_

$$\begin{array}{l} c_{\infty }=\frac{\alpha_{c}}{\alpha_{c}+\beta_{c}};\;\tau_{c}=\frac{1}{\alpha_{c}+\beta_{c}};\\ \alpha_{c}=0.2857(V+34-\sigma )\text{​}/\text{​}(1-\exp(-0.1(V+34-\sigma )));\\ \beta_{c}=3.57\exp((-V-44+\sigma )\text{​}/\text{​}80); \end{array}$$

• ***I***_**Ks**_

$$\begin{array}{l} d_{\infty }=\Gamma (V,34,6.5);\;\tau_{d}=50\text{ms}\\ e_{\infty }=\Gamma (-V,-65,6.6);\;\tau_{e}=200+220\Gamma (V,71.6,6.85);\\ f_{\infty }=\Gamma (-V,-65,6.6);\;\tau_{f}=200+3200\Gamma (V,63.6,4); \end{array}$$

• ***I***_***h***_

$$\begin{array}{l} t_{\infty }=\Gamma (-V,-45,5.5);\\ \tau_{t}=\frac{1}{\left(\exp(-14.59-0.089V)+\exp(-1.87+0.0701V)\right)}; \end{array}$$

where ρ and σ are parameters for the fine tuning of the action potential threshold of the model; and $\Gamma (X,Y,Z)=\frac{1}{1+\exp(-(X+Y)/Z)}$.

The equations were numerically solved with a Runge-Kutta6(5) variable step method with a maximum error of 10^−13^. In all the simulations presented in this paper ρ = 0.6 and σ = 1, unless another value is specified in the simulation description to change the excitability level in different regions of the network. The initial conditions were selected randomly from a set of 10,000 coherent values for all the dynamic variables (10,000 different final conditions in simulations of a single cell) and the stimulus injected current (*I*_inj_ in Equation 1), unless a specific value is indicated to implement external stimuli, was selected randomly between 0.0 and 0.35 μ A/cm^2^.

### 2.2. Network model

To simulate the IO network we have built two-dimensional networks of 50 × 50 IO neurons connected with gap junctions among close neighbors. The term *I*_elec_ in Equation (1) denotes the current received by each neuron through these connections. Therefore, *I*_elec_ = *g*_*c*_ ∑_*i*_ (*V* − *V*_*i*_), where index *i* runs over the neighbors of each neuron and *g*_*c*_ is the electrical coupling conductance. The number of electrically coupled neighbors varied from 4 to 12. We imposed periodic boundary conditions within the network to avoid border effects.

**Figure 3:**
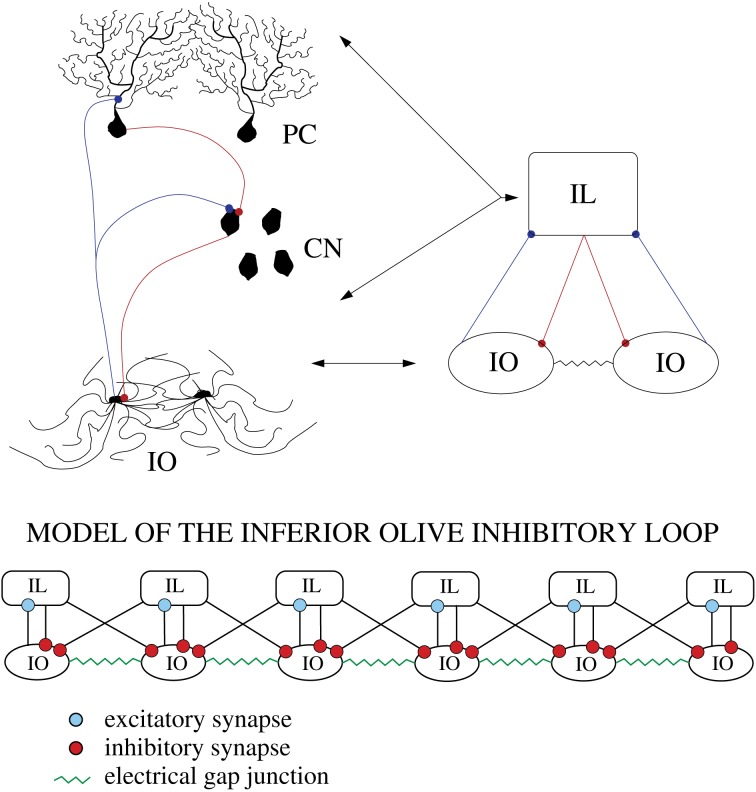
**Schematic representation of the cerebellar inhibitory loop (IL)**. To test the effect of the IL in the IO dynamics we model it with a simple bidimensional network of integrate and fire (IF) units connected to the IO network preserving the topology, i.e., neighbor cells in the IO network sent and received connections to/from neighbor cells in the IL layer.

With this network topology we simulate the autonomous behavior of the IO network. However, as Figure 1 illustrates, the IO forms part of an IL with the deep cerebellar nuclei and the PCs of the cerebellar cortex. To test the effect of this inhibition, we implemented a simplified model of the cerebellar IL that takes into account the timing of these inhibitory inputs omitting the details of the cerebellar neurons and circuits. The inhibitory connections were modeled without the detailed implementation of the cell types involved in the IL (PCs and cerebellar nuclei). The action of the cerebellar IL was built through an inhibitory feedback in the IO networks from a bidimensional network of simple integrate and fire (IF) neurons. Each IF neuron was connected to the IO network bidirectionally as depicted in Figure 3 in one dimension for simplicity. These connections preserved the topology of the IO network, i.e., neighbor cells in the IO network sent and received connections to neighbor cells in the IF layer. The connection probability was 75% in both directions. Each IF neuron took into account whether a group of neighbor IO neurons (up to 10 neighbor cells) had a synchronous spiking event (in a time window of 5 ms), if so, the IF cells evoked a delayed IPSP (10 ms) back to a cluster of up to 10 IO neurons. Then the IF neuron had a refractory period where it could not fire for a short time (10 ms). Inhibitory synaptic currents from the action of the integrate and fire neurons were implemented using the model and parameters described in Destexhe et al. (1994) (ḡ_syn_ = 0.1 nS), and were added into the term *I*_syn_ of Equation (1).

### 2.3. Graphical representation of the spatio-temporal patterns

The spatio-temporal patterns generated by the IO networks consist of propagating wave fronts of spiking activity that can remain bounded in a region of the network. To illustrate the propagating waves of activity in the simulations, we have generated movies of square-shaped networks to represent their evolving dynamics. Each point in the 50 × 50 square represents the evolution in time of the activity of a given neuron within the network. The neural activity is represented with a color scale, where warm colors (red) correspond to neurons with a membrane potential over the spiking threshold (around −47 mV in our model) and cool colors (blue) correspond to hyperpolarized neurons. Intermediate colors represent subthreshold activity. Regions with the same color in the movies have synchronous behavior. Although in the paper we provide snapshots of the evolution of the network activity, the described phenomena are better appreciated in the movies included as supplementary material. To better appreciate the dynamics in slow motion, the temporal scale in the movies does not correspond to the neuron time in ms. The videos are generated at a 25 Hz frame rate and each frame corresponds to 0.5 ms in neuron time.

### 2.4. Wavelet analysis of the patterns

In one dimensional signals, spectral methods are suitable for the detection of rhythms present in the signal. However, in higher dimensions, the coefficients produced by the multidimensional Fourier transform are hard to interpret as they present a number of artifacts not directly related with the behavior of the signal, but to sampling (aliasing effects) or border conditions. Thus, to characterize quantitatively the localized spatio-temporal patterns of the IO network models we did not use spectral methods. Instead, we propose the study of the IO spatio-temporal patterns as a sequence of images evolving in time by means of a wavelet based compression scheme. Wavelet based techniques have proven to be a useful tool for signal analysis (Mallat, 1999) and, in particular, for the study of images or sequences of images (Stollnitz et al., 1996). Unlike the Fourier transform coefficients, where the “frequency” content of the signal cannot be localized in time (or space), the wavelet transform coefficients are determined both by a resolution component and a time (or space) component and, therefore, they represent the resolution content at a given portion of the original signal. The number of coefficients of the wavelet transform that are higher than a given threshold, or alternatively, those that comprise a given percentage of the total energy of the signal, characterizes the whole complexity of the signal. Wavelet based compression schemes are based on this complexity. On one hand, when the number of wavelet coefficients larger than a fixed threshold is small, the corresponding signal can be represented only with a few low resolution components and high compression can be achieved without highly distorting the original data. On the other hand, if the number of coefficients larger than the fixed threshold is high, we have a complex signal, so we will need both high resolution (details) and low resolution components to represent it and, therefore, low compression can be performed. Wavelet based compression schemes have been used, for example, for one dimensional signal segmentation through “wavelet probing” techniques (Deng et al., 1993).

The Wavelet Transform (WT) is related with multiresolution analysis and presents a hierarchical structure that is particularly suited for fast numerical algorithms (Daubechies, 1992). In particular, the multiresolution process allows the computation of the coefficients of the WT by means of the Discrete Wavelet Transform (DWT) with a low computational cost. The two dimensional wavelet transform has been used for image compression, as it presents high compression levels and a low computational cost (*O*(*w* × *h* × *t*) vs. *O*(*w* × *h* × max{log(*w*) × log(*h*)} × *t*) for similar compression schemes based on spectral techniques; where *w* and *h* are the image width and height in pixels, respectively, and *t* is the number of images). Briefly, the idea behind the image compression techniques based on the WT is that wavelet coefficients that correspond to parts of the image that are smooth have a small value (low spatial complexity), in contrast with complex images that present a low number of small parameters of the two dimensional WT and their compression ratios are lower.

Our metric to characterize the IO spatio-temporal patterns is based on the previous compression technique. The method consists in considering the spatio-temporal patterns generated by our IO models as sequences of images and estimating the compression rate of each of them by calculating the number of DWT coefficients higher than a given threshold. In this way, we translate the spatio-temporal pattern to a new one dimensional signal, *C*(*t*), which represents the evolution in time of the spatio-temporal pattern *complexity*. As a first step in the characterization, a two-dimensional basis was generated by direct Cartesian product of the one-dimensional Haar basis (Stollnitz et al., 1996). Then, we calculated the two dimensional non-standard DWT for each frame of network activity and counted the number of coefficients, *C*(*t*), that were larger in absolute value than a given threshold (*th* = 1 in the simulations shown here). The new one dimensional signal provides a useful characterization of the spatio-temporal patterns in which both the frequencies and the spatial complexity can be discussed. At a given time *t*, a high value for *C*(*t*) means that the network has a complex spatial structure, while a low value indicates a uniform space (synchronized activity). The time evolution of *C*(*t*) provides information about the frequency of the spatio-temporal patterns.

### 2.5. Wave front propagation characterization

The IO wave fronts in our simulations have the shape of circles or arcs centered at a given region and propagating through the network. To characterize the evolution of these wave fronts, we have developed an algorithm for the detection of arcs of propagating spiking activity through the analysis of each video frame. Several methods have been developed for the detection of circles in images, most of them based on the Hough Transform (Duda and Hart, 1972). The Hough transform provides accurate results as long as the circle radius is known, and the image has low noise and low density of edge pixels (pixels with value 1 in a binary representation of the image). Furthermore, the Hough transform requires a high amount of memory and computational resources as a non-linear optimization process is involved in the method.

Our method for detection of arcs is based on the idea that a circle is completely determined by the position of three edge pixels in the image representing a given frame of IO network activity. To apply this algorithm, we convert the membrane potential time series to binary time series where 0 means that the neuron is under the firing threshold, and 1 that it is over the threshold. The algorithm iteratively searches for a set of three edge pixels in the frame *F*. Once a set of three edge pixels is found [*p*_1_ = (*x*_1_, *y*_1_), *p*_2_ = (*x*_2_, *y*_2_), *p*_3_ = (*x*_3_, *y*_3_)], the center of the unique circle that contains them is calculated in the following way:

- The general equation of the circle can be written as *x*^2^ + *y*^2^ + *Ax* + *By* + *C* = 0 with center at the point $p=-\frac{1}{2}(A,B)$ and radius $r=\sqrt{C-A^{2}-B^{2}}$. Then the three edge pixels coordinates are used in the previous equation, obtaining the following linear system:
  $$\begin{array}{l} x_{1}{}^{2}+y_{1}{}^{2}+Ax_{1}+By_{1}+C=0\\ x_{2}{}^{2}+y_{2}{}^{2}+Ax_{2}+By_{2}+C=0\\ x_{3}{}^{2}+y_{3}{}^{2}+Ax_{1}+By_{3}+C=0 \end{array}$$
  The values *A, B, C* can now be obtained by methods such as Gauss elimination or Cramer's rule. If these methods cannot solve the system, then the three points lie on a straight line.
- Once the center *p* is obtained, we create a image mask *F*′ with the same size of frame *F* that contains just the plot of the circle with center *p* and radius *r* obtained in the previous step. We then count the number of edge pixels in the mask, and perform a logic *AND* (*F* ∩ *F*′) operation between the original frame *F* and the mask *F*′.
- We count the number of the resulting edge pixels from the previous operation and we consider that there exists a circle in *F* that contains the three points if that number is larger than a given fraction of the edge points in the mask *F*′.

This procedure is repeated until all different circles are found in frame *F*. By analyzing the evolution of centers at different time frames, and the change in the value of the radii of the arcs, we can decide whether each center is a source or a sink.

## 3. Results

### 3.1. Origin and propagation of the spatio-temporal patterns

#### 3.1.1. Gap-junction mediated quasi-synchronized IO activity

*In vitro* experiments demonstrate that IO cells generate spatio-temporal patterns of network activity (Devor and Yarom, 2002b; Leznik et al., 2002; Leznik and Llinás, 2005). In our analysis of these patterns, we first discuss the spontaneous activity of autonomous IO network models without any external stimuli. We have built two-dimensional networks of 50 × 50 IO model neurons connected with gap junctions among close neighbors (see section 2.2 for details). The parameters of the IO cells were set so that they could generate subthreshold oscillations and spiking activity (section 2.1). Different simulations were performed varying the magnitude of the electrical coupling conductance among neurons and the number of electrotonically coupled neighbors.

**Figure 4:**
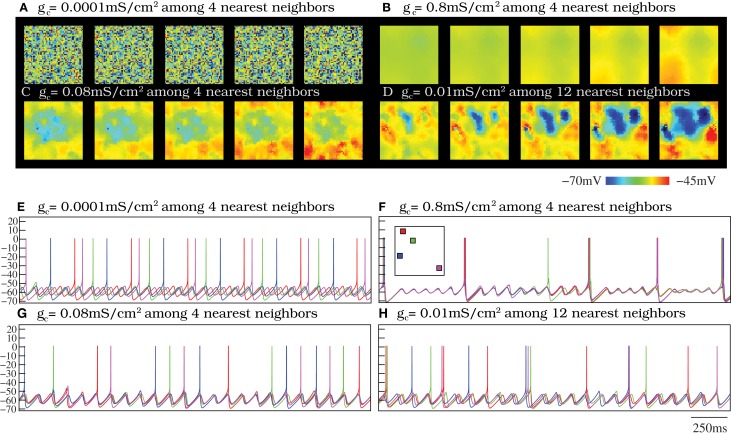
**Spontaneous spatio-temporal patterns generated by the IO network models. (A–D)** Respectively, snapshots of Movies S1–S4. These panels illustrate the patterns of activity of four 50 × 50 IO networks with different connectivity settings: weak coupling **(A)**, strong coupling **(B)**, moderate coupling **(C)**, and weak coupling extended to further neighbors **(D)**. Sequences develop in time from left to right with a 3 ms time interval between frames. Regions with the same color have synchronous behavior. Color bar maps the membrane potential. When the coupling is moderate (panels **C** and **D**), there exist well-defined spatio-temporal patterns of spiking activity traveling over the network. However, when the coupling is either too weak or too strong these spatial structures do not appear. **(E–H)** Membrane potential time series of four randomly chosen neurons from the IO networks whose activity is represented in panels **(A–D)**. Units are mV. The inset in panel **(F)** shows the approximate location of these neurons in the two-dimensional network. The color code matches the time series of each cell. When the strength of the coupling is weak **(E)**, there is not synchrony among cells (cf. panel **A**). However, as the coupling increases, either with moderate electrical conductances or a more extensive connectivity (panels **G** and **H**, respectively), the subthreshold oscillations tend to synchronize. Note the small phase shifts in the quasi-synchronized subthreshold oscillations in these cases. These transient small phase shifts create the spatio-temporal patterns observed in panels **(C,D)** and the corresponding activity movies in the supplementary material. As the strength of the coupling grows **(F)**, the global spiking frequency decreases and, if the electrical coupling is high enough, the individual activity of the cells is completely synchronized (cf. panel **B**).

In a neural media with the features of the IO, currents arising from incoming inputs are invested to increase/decrease the excitability level of each unit and to be shared among neighbors through the diffusive coupling. To address the effect of the strength of the electrical coupling in the spatio-temporal activity of the IO network, we discuss four representative cases illustrated in Movies S1–S4: weak coupling, strong coupling, moderate coupling and weak coupling extended to further neighbors. Sequences of network activity in the form of snapshots of these movies are shown in Figures 4A–D. Sequences develop in time from left to right with a time interval between frames of 3 ms. Both in the videos and in the snapshots, the level of activity of each neuron is represented with the color scale described in section 2.3: warm colors correspond to neurons with a membrane potential over the spiking threshold and cool colors correspond to hyperpolarized neurons.

In these four representative cases, we observe that the activity of the autonomous IO network model strongly depends on the magnitude of the electrical coupling. As expected, when the coupling between close neighbors is too weak, i.e., *g*_*c*_ < 0.01 mS/cm^2^, the IO neuron activity is nearly independent and thus no coherent patterns are formed (see Movie S1 and the corresponding snapshots in Figure 4A). Strong coupling, *g*_*c*_ > 0.7 mS/cm^2^, induces almost total synchronization and also avoids the formation of spatial structure in the patterns, as seen in Movie S2 and Figure 4B. However, networks with moderate values of the coupling always show evolving spatio-temporal patterns (see Movie S3 and Figure 4C). The spatio-temporal patterns consist of transient spiking activity wave fronts that propagate throughout different regions of the IO network. Physiological experiments have revealed that each IO cell can be coupled to a large number of neighbors (Devor and Yarom, 2002b; Hoge et al., 2011). This situation corresponds to our fourth representative case. The simulations show that increasing the extent of the connections has a similar effect in the network dynamics as an increase in the coupling strength. As an example, Figure 4D shows the spatio-temporal patterns corresponding to an IO network with electrical coupling among 12 nearest neighbors with *g*_*c*_ = 0.01 mS/cm^2^ (see also Movie S4). Note that a network with the same coupling strength but electrical connectivity just among four close neighbors displays nearly independent activity (not shown here). Thus, increasing the number of connections among cells in the IO network model leads to equivalent dynamics as those generated with less connections but larger coupling strength (cf. Figures 4C,D).

**Figure 5:**
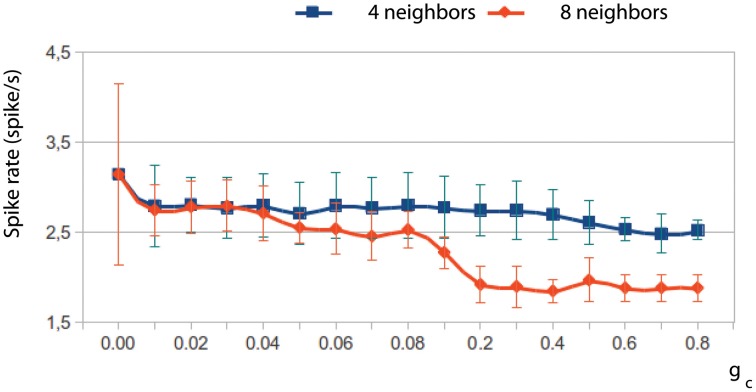
**The electrical coupling decreases the frequency of the global spiking behavior**. The figure shows the evolution of the average firing rate of two IO network models with an electrical coupling among 4 and 8 neighbor cells, respectively, as a function of the coupling strength. Stronger electrical coupling reduces and regularizes the spiking frequency. This phenomenon occurs both increasing the coupling strength and the number of connections among neighbors.

The network dynamics is better understood by looking at the membrane potential of single neurons together with the collective activity shown in the movies. Figures 4E–H plot the membrane potential time series of four representative neurons within the IO networks whose spatio-temporal activity is shown in panels **(A–D)**, correspondingly. The analysis of panels in Figure 4 and the activity movies shows that the patterns arise from small transient phase shifts in the quasi-synchronized subthreshold oscillations for moderate values of the electrical coupling [see the time series of panels **(G)** and **(H)** in Figure 4, and compare them with panels **(E)** and **(F)**]. The occurrence of a spike induces new phase shifts and fast propagating waves that shape the patterns within the quasi-synchronized (period locked) subthreshold activity. However, when the coupling is too weak, the spatio-temporal patterns are absent (see Movie S1 and Figure 4A) since, as can be observed in Figure 4E, the activity of each IO cell is nearly independent because the low electrical current cannot provide coherence to the subthreshold oscillations. Strong coupling also avoids the formation of a spatial structure in the patterns since all the neurons are almost synchronized and follow each other (see Movie S2 and Figure 4F). Higher values of the coupling strength increase the synchronization level but diminish the frequency of the global spiking behavior (cf. Figures 4A–C). Figure 5 quantifies this decrease by showing the average firing rate of the IO network model as a function of the coupling conductance. Stronger electrical coupling has a shunting effect that reduces the excitability of the neurons. Similarly, a larger extent of the electrotonic connections also decreases the firing rates for a strong enough coupling (see Figure 5).

Thus, the autonomous IO network model with the topology discussed above and moderate values of the electrical coupling is able to generate well-defined spatio-temporal patterns based on quasi-synchronized subthreshold activity. The patterns consist of transient propagating wave fronts of spiking activity that can remain bounded in a region of the network. In all the cases discussed so far, the degree of synchrony among cells changes as a function of the coupling conductance although the frequency of the subthreshold oscillations remains nearly constant.

**Figure 6:**
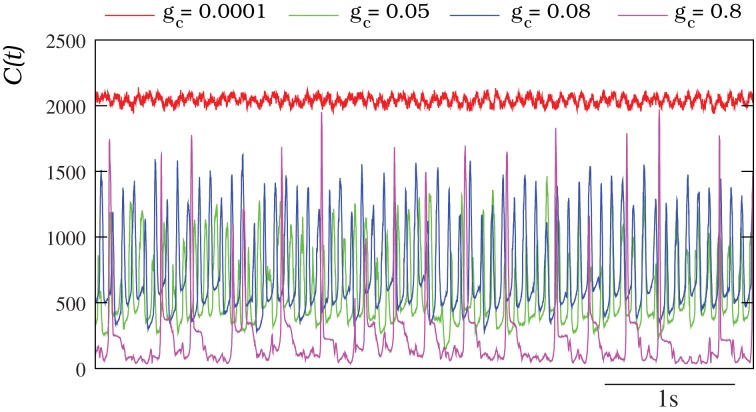
**Characterization of the IO spontaneous activity with the DWT**. In all the cases plotted, each IO neuron in the network is connected to four nearest neighbors with the electrical conductance indicated in each trace. Units for the connectivity are mS/cm^2^. The higher the number of DWT coefficients, the more complex is the spatial organization of the pattern reflecting the absence of coherence in the IO network. For a connectivity *g*_*c*_ = 0.0001 mS/cm^2^, *C*(*t*) remains high all the time. This indicates that the individual activity of each neuron is independent (see Movie S1 and Figure 4A). The oscillations observed in the red trace correspond to the homogeneous subthreshold activity. The opposite case, a low number of DWT coefficients, corresponds to a strong coupling (in the example *g*_*c*_ = 0.8 mS/cm^2^), where the activity of each neuron is almost completely synchronized (see Movie S2 and Figure 4B). The peaks in the magenta trace correspond to the generation and propagation of a spike over the whole network. Finally, when the coupling is moderate (*g*_*c*_ = 0.05 mS/cm^2^ and *g*_*c*_ = 0.08 mS/cm^2^) the evolution of the DWT coefficients shows the evolving spatio-temporal patterns over the same dominant frequency of the subthreshold activity.

The characterization of the spatio-temporal patterns generated by the autonomous IO network models with a discrete wavelet transform analysis (DWT, see section 2.4) corroborates these results. Figure 6 shows the evolution of the number of DWT coefficients for different simulations of autonomous IO networks (spontaneous activity). The red traces correspond to a network with very small coupling among the IO cells. The number of DWT coefficients remains high during the simulation revealing a complex spatial structure in the patterns (i.e., independent single neuron activity). Nevertheless, the homogeneous frequency of the subthreshold oscillations is captured by the DWT analysis. The magenta trace shows the opposite case, a network with a high electrical coupling showing a high degree of synchronization (no complexity in the spatial structure and thus low number of DWT coefficients), only broken briefly at each spike event [see Movie S2 and Figure 4B]. The other two traces (green and blue) correspond to a moderate value of the coupling where the evolving spatio-temporal patterns can be observed with the dominant frequency of the quasi-synchronized subthreshold oscillations. Note the sustained broad range (~200–1500) in the number of DWT coefficients characterizing these patterns.

#### 3.1.2. Activity phase locks in the absence of subthreshold oscillations

**Figure 7:**
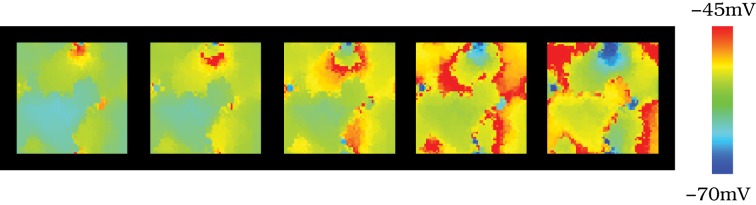
**Spatio-temporal patterns in the absence of subthreshold oscillations**. Snapshots of Movie S5. The figure illustrates the spatio-temporal patterns observed in a network model of 50 × 50 neurons electrically connected to their four nearest neighbors with *g*_*c*_ = 0.05 mS/cm^2^. The individual dynamics in this case does not contain subthreshold oscillations, just spiking activity. In the simulation illustrated here we achieve this behavior by applying a constant current injection (*I*_inj_) in the whole population (Figure 2). Sequences are equivalent to those shown in Figure 4, developing in time from left to right with a time interval between frames equal to 3 ms. When the subthreshold oscillations are not present, the spatial topology of the patterns remains nearly constant.

To investigate to what extent the presence of the subthreshold oscillations influences the structure of the patterns, we also simulated autonomous networks of tonically spiking neurons without subthreshold oscillations. This behavior can be easily induced in the model by adjusting the spiking threshold through the kinetics of the ionic channels of the model (parameter σ) or by applying a constant current injection (*I*_inj_) in the whole population (see section 2.1). When the subthreshold oscillations were not present, the spatial topology of the patterns remained nearly constant (see Movie S5 and Figure 7 with snapshots of the evolution of the network activity in this situation). The fixed spatial organization of the patterns is due to a high degree of sustained phase-locking among neighbor units in the absence of subthreshold oscillations. Movie S5 shows that the propagation of the wave fronts is faster over this nearly fixed spatial shape. In all our simulations, the characteristic spatial structure of the patterns changed significantly in time only when subthreshold oscillations were present in the model.

### 3.2. Rhythm encoding and coordination

The simulations described so far implemented neurons with spontaneous spiking activity over the subthreshold oscillations. A major point of interest in this study was the analysis of the response of the IO network model to stimuli that could induce different coherent spiking frequencies in the IO neurons. The single neuron model can generate different spiking frequencies depending on the current injection (*I*_inj_). The spiking frequencies are commensurate with the subthreshold oscillation frequencies up to the tonic firing (see section 2.1 for details).

#### 3.2.1. Coexistence of stimulus induced rhythms in the IO media

**Figure 8:**
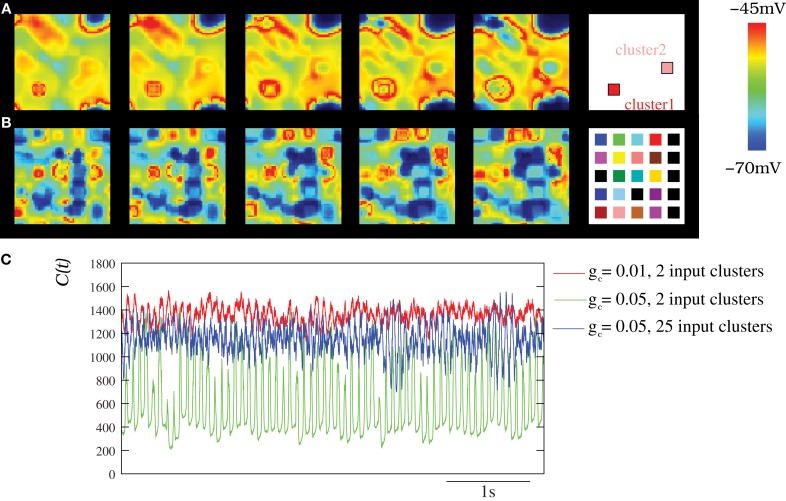
**Spatio-temporal patterns induced by external stimuli in the IO network models. (A–B)** Snapshots of Movies S6 and S7. Sequences develop in time from left to right with a time interval between frames of 3 ms. The snapshots illustrate how several structures with different frequencies can coexist simultaneously in the network when several stimuli are present. The number of connected neighbors in the IO network is eight with *g*_*c*_ = 0.05 mS/cm^2^. Stimuli are introduced in the IO network by means of a constant current injection in different clusters of neighbor neurons (see the text). Panel **(A)** shows the activity of a network with two different clusters of 6 × 6 cells each with different stimuli (*I*_inj1_ = 0.75 μA/cm^2^ and *I*_inj2_ = 0.25 μA/cm^2^). Panels **(B)** shows the activity of a network with 25 different clusters of 6 × 6 cells with *I*_inj_ distributed over 0.1–0.75 μ A/cm^2^. The right panels display the approximate positions of the clusters within the network. Colors represent different current injections, and thus different spiking frequencies in these clusters. **(C)** Characterization of the IO activity with the DWT when the IO network received different external stimuli. The stimuli induces the coexistence of different spatio-temporal patterns with different frequencies and spatial organization (see Movies S6 and S7), which is reflected in a more complex evolution of the DWT coefficients whose shape characterizes the spatio-temporal pattern (cf. with Figure 6). Thus, the stimuli are encoded in these coexisting and coordinated spiking rhythms commensurate with the subthreshold oscillations. In all cases, each IO neuron is connected to its eight nearest neighbors with the electrical conductance indicated in each trace. Units are mS/cm^2^. Red trace corresponds to a network with 2 input clusters of 36 IO neurons in which an injection of 0.25 μA/cm^2^ and 0.75 μ A/cm^2^ was applied. Green trace corresponds to the same network but using a stronger coupling. Blue trace shows *C*(*t*) for a network with 25 clusters of 25 neurons each with different stimuli distributed over 0.1–0.75 μA/cm^2^.

To study the spatio-temporal patterns induced by stimuli to the IO network model, we performed simulations where different external currents were injected in different clusters of neighbor neurons. The stimulus clusters are surrounded by neurons that have no stimuli. The stimulus induces a higher spiking frequency in the neurons of these clusters while sustaining a similar subthreshold oscillation, as compared to the neurons without stimuli. The larger the input current, the higher the spiking frequency. Rhythms induced by the stimuli could then be observed in the network of IO neurons. As an example, Movie S6 shows the spatio-temporal patterns produced in an IO network model when two external stimuli evoking two different spiking frequencies were applied to two clusters of 6 × 6 cells within the network. Figure 8A displays snapshots of this movie showing the coexistence of wave fronts with different spiking frequencies. The right panel in this figure indicates the approximate location of each cluster in the network. The coherent wave fronts originate in the regions with stimuli and generate the spatio-temporal patterns. The spatial scale of the patterns evoked by stimuli in the IO network depends on the frequency of response of the clusters (normal dispersion) and the strength of the coupling. Multiple spatio-temporal structures with different spiking frequencies may coexist simultaneously in the IO networks. For example, Movie S7 (and the corresponding snapshots in Figure 8B) corresponds to a simulation with 25 clusters of 6 × 6 neurons each with different stimuli.

Figure 8C shows the wavelet analysis of three representative examples of networks with several coexisting frequencies of oscillations induced by stimuli. In all the traces, the frequency of the subthreshold oscillations can be observed. However, the spiky waveform indicates the presence of multiple coexisting frequencies. This can be better noted in the blue trace corresponding to the evolution of the DWT coefficients for the network with 25 input clusters. It is important to emphasize that any input to the IO clusters is encoded into a spiking frequency that is commensurate with the subthreshold oscillations, which results in a coordinated network activity.

#### 3.2.2. Sort term memory through stimulus reverberation

In the IO network simulations we observe that the stimulus induced spatio-temporal patterns can survive for several seconds after the excitation is over. This *stimulus reverberation effect* depends on the coupling strength in the network and is illustrated in Movie S8. In the simulation shown in this video, initially the network dynamics evolves freely as in the previously studied autonomous IO networks. Then, the external stimulation starts (at instant 0:40 in the movie) and lasts for 2 s (until the instant 2:00 in the movie). During this interval, we apply two external stimuli to two different clusters of 6 × 6 cells. Note that from the beginning of the stimulation, stepwise, the stimulated clusters become the two principal sources of the spatio-temporal patterns. When the external stimulus is over, this behavior continues for a long period and the IO network generates the same wave fronts that were induced by the stimuli.

**Figure 9:**
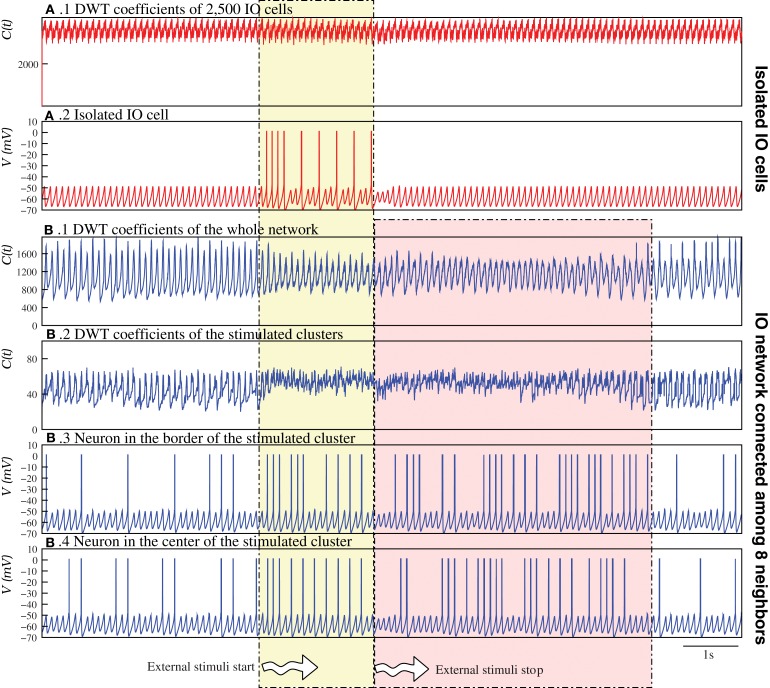
**Stimulus induced spatio-temporal patterns can survive for a long time after the end of the stimulation**. The figure illustrates how stimulus induced spatio-temporal patterns reverberate in the IO network models. In the case of a “network” of 2,500 nearly isolated cells (*g*_*c*_ = 0.00001 mS/cm^2^) **(A)**, in a simulation first without stimuli, then during the stimulation of two clusters of 6 × 6 cells (*I*_inj1_ = 0.75 μA/cm^2^ and *I*_inj2_ = 0.65 μA/cm^2^), and finally without any stimulation again, the DWT coefficients **(A.1)** reveal a complex spatio-temporal structure in the network activity, even during the stimulation. No changes in the global dynamics appear when the two clusters are stimulated, although the individual activity of the stimulated cells changes during this interval **(A.2)**. Focusing on the individual activity of these cells, we observe that when the stimulus begins the neuron starts generating spiking activity. In this case, when the stimulus is over, the neuron goes back immediately to the autonomous activity. **(B)** In contrast, when the neurons have moderate coupling, the change in the network dynamics due to the excitation lasts for several seconds after the stimulation (see Movie S8). The DWT coefficients shows this effect, both in the whole network dynamics **(B.1)** and in the clusters of stimulated neurons **(B.2)**. Panels **(B.3,B.4)** show the changes in the activity of a neuron at the border and at the middle of a stimulated cluster, respectively.

Figure 9 analyzes in detail this phenomenon by comparing an IO network of nearly isolated cells (panels **A.1, A.2**) and a network with moderate coupling (panels **B.1–B.4**). To highlight the reverberant effect, for this analysis the parameters of each individual IO cell were set to generate just subthreshold oscillations without spiking activity in isolation. Therefore, the spiking activity without an external stimulus is a network effect (cf. the neuron activity before the stimuli in Figures 9A.2,B.3). Figures 9B.1,B.2 show, respectively, the evolution of the DWT coefficients of the whole network and the stimulated clusters in the simulation with moderate coupling. This DWT analysis shows that the global network dynamics changes when the external stimuli are applied. Note that the change due to the excitation lasts for several seconds after the stimulation is over, and then the network goes back to the autonomous activity. Conversely, in the network with very weak coupling (Figures 9A.1,A.2), the change in the dynamics induced by the external stimulus is not sustained when the stimulus is over.

#### 3.2.3. Activity source-sink phenomena

**Figure 10:**
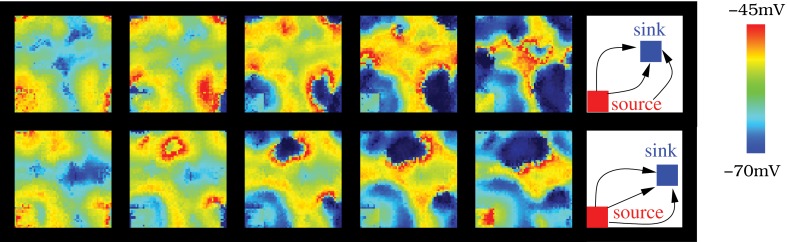
**The source-sink phenomena appears in the IO network model when a cluster of neurons is set to have a high rate of spiking activity while another is set in a subthreshold oscillatory regime**. In the networks illustrated in the figure, the number of connections among the nearest neighbors is 12 with *g*_*c*_ = 0.01 mS/cm^2^. Sequences develop in time from left to right with a time interval between frames of 10 ms. Right panel shows the approximate location of the source (cluster with highly excitable neurons, *I*_inj_ = 0.5 μA/cm^2^) and the sink, (cluster with low excitable neurons, σ = 2 and *I*_inj_ = 0 μA/cm^2^). The difference between the top and bottom panel is the location of the sink cluster (the source is the same in both cases).

Another remarkable feature arising in the IO model networks is the activity *source-sink* phenomena when at least two specific clusters of neurons are present: one cluster with a higher rate of spiking activity than the average population and the other with no intrinsic spiking activity (subthreshold oscillating neurons) or low excitability. In this situation, the wave fronts generated in the cluster with high excitability (source) travel to the cluster with low excitability (sink). To identify the sources and sinks we use the algorithm described in section 2.5 which can characterize the wave front propagation. To apply this algorithm, first we convert the membrane potential time series to binary time series where 0 means that the neuron is under the firing threshold (−47 mV), and 1 that it is over the threshold. Then, to identify the wave front sources and sinks, we search for arcs centered in a given region and analyze the evolution of their mean radius. In the activity sources, the arc radius grows; while in the sinks the radius decreases. Table 2 shows the result of this analysis in two simulations where the source and the sink are located in different regions. In both cases, each IO neuron is connected to 12 neighbors with electrical coupling *g*_*c*_ = 0.01 mS/cm^2^. Figure 10 displays snapshots of these simulations. The approximate location of each cluster is shown in the right panels. In these simulations, more than 50% of the wave-front arcs whose radii increase are centered in the cluster with a higher spiking rate. Near 60% of the arcs whose radii decrease travel to the cluster of subthreshold oscillating cells.

**Table 2:** **Characterization of the wave front propagation traveling from the source to the sink**.

	**Cluster**	***t***_**1**_	***t***_**2**_	***t***_**3**_	***t***_**4**_	***t***_**5**_
*S*_*A*_	Source	2.6 ± 0.5	6.8 ± 0.8	10.0 ± 1.3	15.7 ± 2.7	–
*S*_*A*_	Sink	–	14.9 ± 3.4	9.6 ± 2.4	3.1 ± 0.7	2.2 ± 0.4
*S*_*B*_	Source	2.3 ± 0.5	6.6 ± 1.0	9.7 ± 1.1	14.4 ± 1.9	–
*S*_*B*_	Sink	–	13.9 ± 2.0	8.2 ± 0.9	3.9 ± 1.2	2.1 ± 0.4

The table shows the evolution of the mean radius of the IO wave fronts in two representative cases to illustrate the activity source-sink phenomena. In a simulation where a cluster of neurons has a higher rate of spiking activity than the average population (C_A_) and another cluster has only subthreshold oscillating neurons (C_B_), the evolution of the mean radius of the arcs with center in these clusters shows that the wave fronts generated in C_A_ travel to C_B_. The table characterizes the wave front propagation corresponding to the two simulations (S_A_ and S_B_) illustrated in Figure 10. The number of connections among the nearest neighbors is 12 with g_c_ = 0.01 mS/cm^2^. Both clusters consist of 6 × 6 neighbor neurons. In the cluster with highly excitable neurons (source) I_inj_ = 0.5 μ A/cm^2^, while in the cluster with low excitable neurons (sink) σ = 2 and I_inj_ = 0 μ A/cm^2^. To calculate the mean radius, the interval from the wave front birth to the wave front death was divided in five subintervals (t_i_) with the same duration. Dashes indicate that no arcs were detected for that interval. Units are dimensionless as the radii were calculated in terms of the number of adjacent neurons covered by a spatio-temporal pattern from a given center detected with the wave front characterization algorithm described in section 2.5.

**Figure 11:**
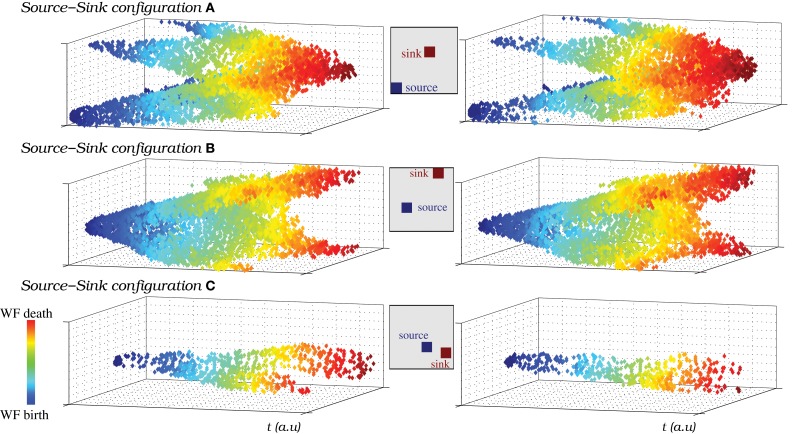
**(A–C)** The source-sink phenomena allows the IO network models to generate spiking wave fronts from different regions and attract them to specific regions. To represent the pathways followed by the spatio-temporal patterns generated in a network model, we plot the IO cells that are over the firing threshold in each moment, from the wave front birth to its death. *y* and *z* axes represent the neuron coordinates in the IO network (50 × 50 square shaped), while *x* axis represents time evolution. Note that time is counted in terms of frames. The color code is used to illustrate the evolution of the wave fronts, blue corresponds to moments near their birth and red to moments near their death. We have selected three different pairs of locations for a single source and a single sink in the same IO network (also shown in Movie S9). A total of six pathways are shown in the figure, two examples for each pathway. The insets indicate the approximate location of the source and the sink in each case. The figure shows that the wave fronts are generated in the regions with a higher rate of spiking activity than the average population (sources), then travel through the IO network in different trajectories depending on the sink location and finally die in this region. Note the effect of the periodic boundary conditions of the network in this representation.

The wave fronts originated in the source generate secondary wave fronts and travel through the IO network following different trajectories depending on the sink location where they finally die. Figure 11 illustrates how the source-sink phenomena allows the IO network models to generate spiking wave fronts in different regions and attract them to specific locations by modulating the excitability of the IO cells. The figure compares the pathways followed by the spatio-temporal patterns produced in a network of strongly coupled cells (*g*_*c*_ = 0.8 mS/cm^2^ among eight neighbors) as a function of the source and sink location. Insets in each panel indicate the approximate position of both regions in each situation. The strong level of coupling facilitates the analysis of the traveling spiking activity since in this case only one wave front is active at a give time. The pathways in Figure 11 correspond to a simulation where both the sink and source location change in time. Note that the trajectories are similar when the source and the sink are in the same position (cf. left and right columns). In particular, they have the same origin and die in the same destination. Movie S9 is a movie of this simulation. Note that, to better show the propagation of spike wave fronts, in this activity movie the color scale changes. Each time the source/sink location changes in the simulation, the new position is pointed out in the video. The spatio-temporal patterns mostly travel from the source to the sink (Figure 11). Nevertheless, the video shows the competition between the global intrinsic IO network dynamics and the source dynamics. This competition allows the generation of wave fronts in a location different from the source traveling to the sink (e.g., the wave front generated in the left-upper corner at instant 0:43 in the movie). Finally, the video also shows that, due to the stimulus reverberation effect, after each change in the excitability of a group of cells, it may exist a short interval where the spatio-temporal patterns do not travel to the sink region (e.g., the first wave front generated after the sink/source location change at instant 0:32). After this adaptation period, the wave fronts are attracted to the new sink.

Different stimulus can shape the presence of sources and sinks in the spatio-temporal patterns of the IO. The ability to attract the wave fronts from one region to another by modulating the excitability can be an important feature for a system with topology preserving connections as those found in the cerebellar circuits.

### 3.3. Effect of the inhibitory loop

As Figure 1 illustrates, the IO is part of an IL with the deep cerebellar nuclei and the PCs of the cerebellar cortex. The terminals of the inhibitory synapses from the cerebellar nuclei are located close to the gap junctions of the IO (Sotelo et al., 1986) and this can produce a transient decoupling of neighbor neurons. The effect of inhibitory synapses into the IO network could in principle destroy the quasi-synchronization of the subthreshold oscillations observed in the previous simulations, and thus destroy or largely affect the dynamics of the spatio-temporal patterns. To test the effect of this inhibition we have performed simulations using a simplified model of cerebellar IL (see section 2.2).

**Figure 12:**
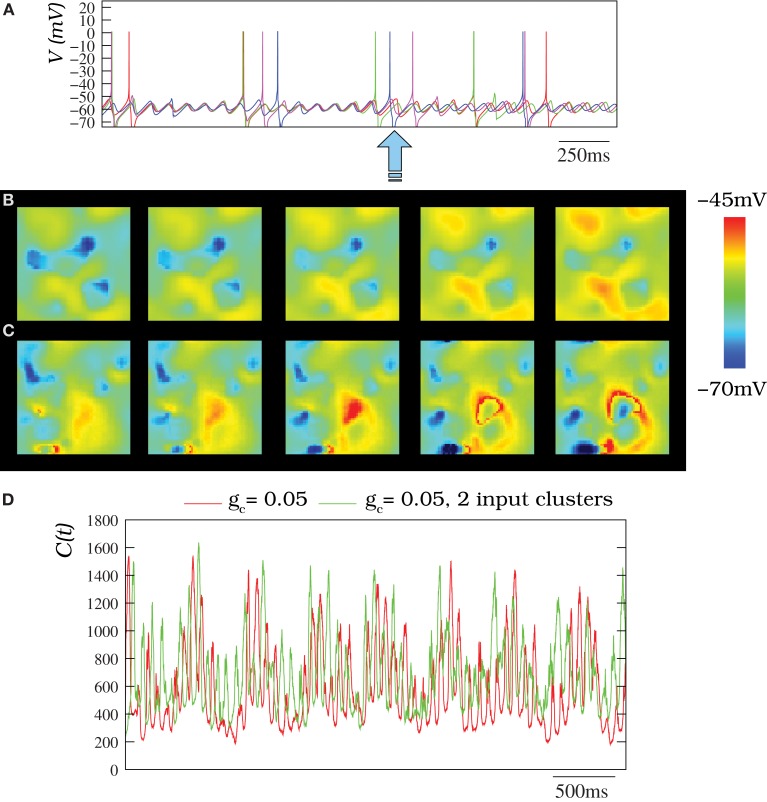
**(A)** Membrane time series for four randomly chosen neurons (the same as in Figure 4) within an IO network model of 50 × 50 units where the inhibitory loop is present. Each neuron is connected to its eight nearest neighbors with *g*_*c*_ = 0.05 mS/cm^2^. In the absent of inhibition, subthreshold activity with a moderate coupling is quasi-synchronized (cf. Figure 4G). However, here, there exist transient desynchronizations induced by the inhibitory feedback from the IL (denoted by the arrow). **(B)** Spatio-temporal patterns observed in IO network model without external stimuli under the modulatory effect of the IL (snapshots of Movie S10). **(C)** Activity of an IO network with two clusters of neurons with an external stimuli (snapshots of Movie S11). In panels **(B,C)** the IO network topology and stimuli clusters are the same described for Figure 8A. Sequences develop in time from left to right with a time interval between frames of 3 ms. **(D)** Number of coefficients of the two-dimensional DWT that are bigger than 1 for different situations where the IL is present. The red trace corresponds to an IO network without stimuli where each cell is connected with its eight nearest neighbors with *g*_*c*_ = 0.05 mS/cm^2^. The green trace corresponds to the same network but with two input clusters of 36 neurons with an injection of 0.25 μ A/cm^2^ and 0.5 μA/cm^2^. Note the IL induces an additional spatial modulation in the IO network activity. Nevertheless, the spatio-temporal patterns generated by the IO network do not disappear.

The presence of inhibitory chemical synapses coming from the IL changed both the spiking frequency and the frequency of the subthreshold oscillations in the IO network simulations. Each synapse induced a transient desynchronization of the subthreshold oscillations among neighbor cells (Figure 12A). The synchronization was recovered later for close enough cells and the spatio-temporal patterns were not destroyed, but received an additional modulation (see wavelet analysis below). Movies S10, S11 illustrate the dynamics of two IO networks where the IL is present: one without stimuli and the other with several stimuli (Figures 12B,C show snapshots of these movies). As in the autonomous networks, several frequencies for the oscillations could also be distributed in different clusters with different stimuli in the presence of the IL (see Movie S11 and the corresponding snapshots in Figure 12C). The inhibitory connections affect the extent of the propagation of the patterns in the network and a larger coupling conductance or number of connections is needed to reproduce the extent of the patterns without the inhibition.

The inhibitory modulation is hardly appreciated by an eye inspection (e.g., see Movies S10, S11), but can be seen in the wavelet analysis. Figure 12D corresponds to the wavelet analysis of networks with the IL. The evolution of DWT coefficients in these networks clearly shows the slow modulatory effect induced by the IL in two networks with *g*_*c*_ = 0.05 mS/cm^2^ in the absent or present of stimuli. Note that the more spiky trace (green trace) corresponds to a network with an external stimuli. Thus, the simulations indicate the IL can introduce an additional modulation in the IO network activity without destroying the patterns.

## 4. Discussion

While the anatomy and physiology of the cerebellar circuits has been studied for more than a century now, the possible roles of the IO are still under discussion. The activity of this neural system has been analyzed mainly at the level of single-cell recordings, from which network properties were then inferred. In particular, electrophysiological and imaging techniques have allowed the direct study of the IO network activity in *in vitro* (Devor and Yarom, 2002b; Leznik et al., 2002; Leznik and Llinás, 2005; Chorev et al., 2007; Hoge et al., 2011) and *in vivo* (Chorev et al., 2007) experiments. However, the dynamical properties of IO networks *in vivo* have not been explored in detail. Two major hypothesis have been proposed about the IO: (1) the learning hypothesis, in which IO activity modifies through long-term depression the cerebellar input and output (Ito, 1982; Kobayashi et al., 1998; Ito, 2005; Swain et al., 2011); and (2) the IO activity contributes to motor control in real time through its intrinsically rhythmic synchronous activity (Welsh et al., 1995; Jacobson et al., 2008). Another proposal brings together these two views and postulates that the major role of the IO is to reduce the firing rate carrying the error signal for cerebellar learning while maintaining its information content (Schweighofer et al., 2004, 2013). All these hypotheses are plausible for this neural system with very rich dynamical properties and likely to be multifunctional.

In this paper we have studied for the first time the IO dynamics using a large scale network with conductance-based models. This type of model is necessary to address the dynamics that arises from the interaction between the spiking activity and the subthreshold oscillations in the context of a diffusive neural media built on gap-junctions. Electrical gap junctions have been suggested as a key factor for the characteristic rhythmic dynamics in the IO network (Blenkinsop and Lang, 2006; Marshall et al., 2007). In our simulations, both the subthreshold oscillations and the spiking activity, propagated through the gap junctions, strongly contribute to the generation of coherent and coordinated spatio-temporal patterns for a large range of coupling strengths. The coordination arises from the subthreshold oscillations that keep a high degree of synchronization due to the extensive electrical connectivity while allowing different spiking frequencies in distinct regions of the IO network.

In the presence of stimuli, different rhythms can be encoded in the spiking activity of the model IO neurons that nevertheless remains constrained to a commensurate value of the subthreshold frequency. Experimental recordings show that subthreshold oscillations in the living IO cells are very precise (Devor and Yarom, 2002a), although their frequency can change at different moments or between different groups of cells (Devor and Yarom, 2002b; Chorev et al., 2007). In this context, the climbing fibers to PCs in the cerebral cortex could carry motor signals beating on the rhythm of the subthreshold oscillations being locally propagated through the precisely timed wave fronts of the IO spiking activity. It is also possible in this system the organization of a context dependent coordination of the spatio-temporal patterns that are coming from different sources. Both these functions could provide, from the commensurability of the different incoming frequencies, a convenient representation of motor rhythms for the next processing levels.

Several transient dynamical phenomena were identified in the simulations of IO networks that can be useful for a precise encoding and coordination of rhythms. The specific properties of the dynamic organization of the IO patterns observed in our simulations can be summarized in the following points:

- Both the characteristic subthreshold oscillations and the spiking behavior of the IO cells are essential for the genesis of the transient spatio-temporal patterns in the network.
- Higher values of the electrical coupling conductance *g*_*c*_ among cells increased the synchronization level and diminished the frequency of the spiking behavior.
- A higher number of electrically coupled neighbors also decreased the frequency of the spiking behavior for a strong enough coupling. In this case the degree of synchrony among cells was higher although the frequency of the subthreshold oscillations remained nearly constant under all these changes.
- The presence of regions with different stimuli could organize clusters of cells (i.e., localized patterns) with different spiking frequencies encoding the stimuli and coexisting in the network at the same time under the coherence and commensurability provided by the subthreshold oscillations. The spatial scale of the patterns evoked by a stimulus depended on the frequency of the response.
- The stimuli induced spatio-temporal patterns can reverberate in the network for long periods after the end of the stimuli. This can be used as a short term memory mechanism in such media.
- The wave fronts of spiking activity can be transported through the network to specific locations by regulating the excitability of different clusters (source-sink phenomena).
- The IL introduces an additional slow modulation in the patterns that can be used for further encoding tasks. In any case, the induced transient desynchronization does not destroy the spatio-temporal patterns.

The spatio-temporal patterns in our simulations were similar to those observed in imaging recording of IO slices reported in Manor et al. (2000), Leznik et al. (2002), and Leznik and Llinás (2005). The large scale modeling of IO networks is a powerful tool to interpret the imaging recordings and to overcome the restricted amount of experiments that can be done in these setups (Varona et al., 2002; Torben-Nielsen et al., 2012). In particular, the models can tackle the study of the effect of the IL arriving from the cerebellar nuclei, which is difficult to assess through *in vitro* experimental recordings. In short, IO network simulations can help us to test hypotheses related to the role of cellular and network processes in the genesis of neuronal spatio-temporal patterns, as well as to understand how the IO oscillations encode and control several simultaneous rhythms.

In the context of the study of spatio-temporal dynamics in brain circuits, an important question is how detailed the single neuron model has to be (Rabinovich et al., 2006c). The answer depends on what we are planning to model, the functions of some brain network or a specific system. In our case, our IO network model had to display subthreshold oscillations, spiking activity and input-specific excitability modulations. As we addressed the effect of the interaction between the subthreshold and spiking transient activity in the IO networks, our study required a model that could describe the generation and propagation of currents during the action potentials. Many morphological and physiological details of the IO neurons and the IL were not considered in the model discussed in this paper, but the fundamental dynamical phenomena observed here does not likely depend on these details.

Synchronization at different levels is one of the most discussed phenomena in relation to neural coding (Engel et al., 1992; Diesmann et al., 1999) and neural dynamics (Chow and Kopell, 2000; Rabinovich et al., 2000b, 2006a; Engel et al., 2001), particularly in the context of electrically coupled neurons (Bennett and Zukin, 2004; Connors and Long, 2004). Sustained or transient phase locks and phase synchronization have been extensively studied both experimentally and theoretically (e.g., see Chow and Kopell, 2000; Rabinovich et al., 2006b; Rabinovich and Varona, 2011; Latorre et al., 2013). Several features of transient spatio-temporal pattern activity are universal for excitable systems of different nature (biological, chemical, physical). For example, the emergence of large-scale spatio-temporal patterns in the form of synchronized spirals is typical for epileptic brains (Stacey, 2012), termo-convection and many other media (Rabinovich et al., 2000a). The results of this work can be generalized beyond IO studies, as the control of wave pattern propagation is a highly relevant problem in the context of normal and pathological states in neural systems (e.g., related to tremor, migraine, epilepsy, etc.) where the study of the modulation of activity sinks and sources can have a potential large impact.

Our modeling has shown important phenomena observed in IO *in vitro* and *in vivo* experiments and produced new predictions regarding the computational properties of this network. IO spatio-temporal patterns demonstrate specific features because the IO is a two-level neural media consisting of subthreshold oscillations and spiking activity in the context of diffusive electrical coupling. These two kinds of activity mutually interact: spikes influence the phase of the subthreshold oscillation and at the same time these oscillations determine the probability of the spikes to occur and coordinate the coherency of large-scale patterns. Together with the inhibitory feedback, such specificity of the IO system demonstrates a unique long-lasting encoding and highly shapeable spatio-temporal patterns that can participate in functions related to timing control, learning and motor memory.

Excitatory inputs to the IO neurons coming from the deep cerebellar nuclei or mesodiencephalic junction, which is innervated by excitatory projection neurons of the cerebellar nuclei (De Zeeuw and Ruigrok, 1994), have not been discussed in this paper. Some of the neurons of the mesodiencephalic junction project directly to motoneurons and interneurons in the spinal cord responsible for motor activity. Interestingly, the olivo-cerebellar loops appears to be topographically organized (De Zeeuw et al., 1998), and they surely react to each of the local spiking frequencies in the IO patterns. This means that if different patterns are clustered in the IO encoding different rhythms, they could be coordinated, transported and controlled through the intrinsic dynamical properties discussed above.

### Conflict of interest statement

The authors declare that the research was conducted in the absence of any commercial or financial relationships that could be construed as a potential conflict of interest.

## Supplementary material

Movie S1 **Nearly independent activity in an autonomous weakly coupled IO network model**. Each neuron is connected to four neighbors with a weak electrical coupling (*g*_*c*_ < 0.001 mS/cm^2^). See section 2.3 for a description of the graphical representation. Note that the time scale does not correspond with the simulation time. For low coupling conductances, the activity of the neurons is nearly independent (see Figure 4E) and the spatio-temporal patterns are absent.

Movie S2 **Synchronized activity in an autonomous strongly coupled IO network model**. The network displayed in this video is equivalent to the network in Movie S1 but with a strong electrical coupling (*g*_*c*_ < 0.8 mS/cm^2^). In this situation, there is almost total synchronization among neurons (see Figure 4F), only broken briefly when spiking behavior occurs, and no spatio-temporal patterns are generated.

Movie S3 **Spatio-temporal patterns of coordinated activity in an autonomous moderately coupled IO network model**. The network displayed in this video is analogous to the network in Movies S1, S2 but with a moderate electrical coupling among cells (*g*_*c*_ < 0.08 mS/cm^2^). In this case, the individual neurons have quasi-synchronized subthreshold activity (see Figure 4G) and the network displays well-defined spatio-temporal patterns consisting of propagating wave fronts of spiking activity from transient phase shifts in the subthreshold oscillations.

Movie S4 **Spatio-temporal patterns in an autonomous IO network model with weak coupling extended to further neighbors**. Each neuron is connected to 12 neighbors with a weak electrical coupling (*g*_*c*_ < 0.01 mS/cm^2^). The effect of increasing the number of connections between neighbors is equivalent to increasing the coupling strength to a moderate magnitude (cf. Movie S3).

Movie S5 **Nearly constant spatial pattern topology in the absence of subthreshold oscillations**. The video shows the activity of an autonomous IO network of tonically spiking neurons without subthreshold oscillations where each unit is connected to four neighbors with a weak electrical coupling (*g*_*c*_ < 0.05 mS/cm^2^). The spiking behavior is induced in all neurons of the network by injecting a constant current (see section 2.1 and Figure 2 for details). In this situation, the IO network generates spatio-temporal patterns, but their spatial topology remains nearly constant (cf. Movie S3).

Movie S6 **Coexistence and coordination of spatio-temporal patterns induced by stimuli**. Activity of an IO network with two external stimuli. The number of connected neighbors is eight with *g*_*c*_ = 0.05 mS/cm^2^. External stimuli are introduced as a constant current injected in clusters of closed neurons. In this case, we consider two clusters of 6 × 6 cells with *I*_inj1_ = 0.75 μ A/cm^2^ and *I*_inj2_ = 0.25 μ A/cm^2^. Figure 8A shows the approximate position of each cluster. The stimulated neurons have a higher spiking frequency. We observe that each stimulated cluster is the source of a wave front with different frequencies. The IO network is able to simultaneously encode several coexisting spiking rhythms.

Movie S7 **Encoding of multiple simultaneous rhythms**. The network in this video is equivalent to the network in Movie S6, but with 25 stimulated clusters of 6 × 6 cells with different current injections. Figure 8B shows the approximate position of each cluster. The effect observed here is the same observed in Movie S6, but with many more coexisting spiking rhythms that are nevertheless coordinated through the subthreshold oscillations. Different current injections in the different clusters are encoded in different spiking rhythms.

Movie S8 **Stimulus reverberation allows short memory mechanisms in the IO models**. Activity movie showing the stimulus reverberation effect in an IO network model where each neuron is connected to four nearest neighbors with electrical coupling *g*_*c*_ = 0.05 mS/cm^2^. Individual neuron parameters in this simulation are σ = 2 and *I*_inj_ = 0.35 μA/cm^2^. Note that parameters are set so that each individual neuron only displays subthreshold oscillations in isolation. The simulation starts with a period without stimulation (from 0:00 to 0:40). After this interval, two clusters of 6 × 6 neurons receive an external stimuli (from 0:40 to 2:00). Here on, the IO network does not receive any external stimulus again. Note that the stimulus induced spatio-temporal patterns survive long after the end of the excitation.

Movie S9 **The source-sink phenomena allows the IO models to attract the wave fronts to specific locations**. Note that in this video the color scale changes with respect to the one used in the rest of IO activity movies. Here, blue color means that the corresponding neuron is under the firing threshold, and red indicates that neurons are firing. To better illustrate the wave front propagation trajectory, no intermediate colors are used. During the simulation, the location of the source and the sink changes. Each change is graphically indicated in the movie. The spatio-temporal patterns originated in the source cluster (high excitability region) die in the sink cluster (low excitability region). Wave fronts are attracted to the changing low excitability regions (cf. Figure 11).

Movie S10 **Modulatory effect of the IL in autonomous IO networks**. Activity movie showing the modulatory effect of the IL in an autonomous IO network model where each neuron is connected to its eight nearest neighbors with an electrical coupling *g*_*c*_ = 0.05 mS/cm^2^ (cf. Figures 6 and Figures 12D, which shows the DWT analysis).

Movie S11 **Modulatory effect of the IL in the presence of stimuli**. Activity movie showing the modulatory effect of the IL in the same network model as in Movie S10 but in the presence of two external stimuli (cf. Figures 8C and Figures 12D, which shows the DWT analysis).
